# Transactions of Gynecological Society, 1878

**Published:** 1879-06

**Authors:** 


### Transactions of Gynecological Society, 1878.

NOTICE.—With Volume III. of Gynecological Transactions, a new style of binding has been adopted, since that which was used on the preceding volumes proved to lack durability so as to be wholly unsuitable for a permanent binding.

Fellows and Subscribers who wish to have Vols. I. and II., uniform with Vol. III., can procure covers for those volumes by remitting 50 cents for each to the publishers, HOUGHTON, OSGOOD & Co.

This new binding is ornamental, and no doubt very durable.

